# High-Dose Tigecycline for Multidrug-Resistant Serratia Infections in Critically Ill Adults: A Systematic Review of Direct and Indirect Clinical Evidence

**DOI:** 10.7759/cureus.116890

**Published:** 2026-09-25

**Authors:** Arnab Choudhury, Ashutosh Chaturvedi, Adil Ashraf, KV Padmaprakash

**Affiliations:** 1 Department of Medicine, Command Hospital (Eastern Command), Kolkata, IND; 2 Department of Critical Care Medicine, Command Hospital (Eastern Command), Kolkata, IND; 3 Department of Gastroenterology and Hepatology, Command Hospital (Eastern Command), Kolkata, IND

**Keywords:** carbapenem resistance, high-dose tigecycline, hypofibrinogenemia, intensive care, multidrug resistance, narrative synthesis, pharmacokinetics, serratia marcescens, systematic review, tigecycline

## Abstract

Evidence for high-dose tigecycline in severe multidrug-resistant Gram-negative infections comes mainly from non-Serratia cohorts. Its value in critically ill adults with resistant Serratia infection is uncertain. PubMed/MEDLINE, CENTRAL, and Consensus were searched up to July 17, 2026. Two evidence sets addressed high-dose tigecycline effectiveness, pharmacokinetics, and safety, and clinical and microbiological evidence for resistant Serratia. Study-level risk ratios were presented without pooling. Searches identified 2,294 records, and 36 studies reported in 37 publications were included. No study reported a verified high-dose regimen in confirmed resistant Serratia infection with a separately extractable organism-specific outcome. Six observational studies used the same high-dose and standard-dose definitions. Fixed 28-day or 30-day mortality risk ratios ranged from 0.34 to 1.30, and all 95% confidence intervals crossed 1.0. Clinical response favored high dose in two studies, with risk ratios of 1.73 (95% confidence interval: 1.22-2.46) and 1.48 (95% confidence interval: 1.08-2.02). Microbiological response favored high dose in two studies, with risk ratios of 1.75 (95% confidence interval: 1.09-2.81) and 1.92 (95% confidence interval: 1.12-3.28). No pooled estimate was calculated. High dose produced a greater fibrinogen decline in one cohort (-3.05 ± 1.67 versus -1.75 ± 1.90 g/L; p=0.038) and more hepatic or platelet abnormalities in another. Direct evidence for high-dose tigecycline in resistant Serratia infection is absent. Indirect studies show possible response benefits without a consistent survival advantage. Treatment decisions should consider infection site, susceptibility, active alternatives, and coagulation risk. By synthesizing both pathogen-specific studies and relevant indirect clinical evidence, this review aimed to provide a more comprehensive assessment of the potential role of high-dose tigecycline in critically ill adults with resistant *S. marcescens* infections.

## Introduction and background

High-dose tigecycline is used when severe multidrug-resistant Gram-negative infection leaves few active options. Comparative and pharmacokinetic studies have assessed intensified dosing, mainly in *Klebsiella pneumoniae* or *Acinetobacter baumannii *[1-9]. Tissue distribution may support selected infection sites, but low plasma exposure is a concern in bloodstream infection. Observational studies have reported fibrinogen decline and other coagulation abnormalities during tigecycline treatment [10-13]. Dose, duration, and cumulative exposure may contribute, but sepsis, hepatic dysfunction, and concurrent drugs can produce similar changes.

*Serratia marcescens* causes bloodstream, respiratory, urinary, and intra-abdominal infections. Resistant isolates have been described in clinical cohorts [14-20], patient-level or outbreak reports [21-26], and surveillance or genomic studies [27-29]. Mixed-organism cohorts often omit Serratia-specific outcomes, and outbreak reports may omit tigecycline dose or duration.

Earlier reviews examined high-dose tigecycline for severe infections or as an alternative for AmpC-producing bloodstream infection [30,31]. They did not answer whether 200 mg loading followed by 100 mg every 12 h improves outcomes in critically ill adults with resistant Serratia infection. This review assessed that question without assigning effects from other organisms to Serratia.

This systematic review was undertaken to critically evaluate the available evidence on high-dose tigecycline for multidrug-resistant (MDR) *Serratia marcescens* infections in critically ill adults. The review was conducted and reported in accordance with established guidance for systematic reviews, including the PRISMA 2020 Statement, Preferred Reporting Items for Systematic Reviews and Meta-Analyses literature search extension (PRISMA-S) recommendations for literature searching, the Synthesis Without Meta-analysis (SWiM) reporting guideline for narrative synthesis, and validated tools for assessing the risk of bias in randomized and non-randomized studies [32-36].

MDR Gram-negative infections continue to pose a major challenge in intensive care, where delayed or ineffective antimicrobial therapy is associated with poor clinical outcomes. Tigecycline remains one of the few agents with activity against many MDR Gram-negative pathogens and is increasingly used as salvage or combination therapy when treatment options are limited. Because standard dosing may not achieve adequate drug exposure in critically ill patients, particularly in severe infections, high-dose regimens have been adopted to improve pharmacokinetic and pharmacodynamic target attainment. Clinical and pharmacokinetic studies have suggested that intensified dosing may enhance treatment efficacy in selected patients, although supporting evidence remains limited [37-40].

*Serratia marcescens* has emerged as an increasingly important nosocomial pathogen responsible for bloodstream, respiratory, urinary tract, and intra-abdominal infections. The spread of carbapenem-resistant and extensively drug-resistant isolates, driven by diverse resistance mechanisms including carbapenemase production, has further reduced therapeutic options and complicated clinical management [41-45]. However, most published data on high-dose tigecycline involve infections caused by *Klebsiella pneumoniae* or *Acinetobacter baumannii*, while evidence specific to resistant *S. marcescens* remains sparse. Consequently, clinicians often rely on indirect evidence when managing these challenging infections.

## Review

Methods

Review Design and Reporting

This systematic review followed PRISMA 2020 [32], PRISMA-S [33], and SWiM guidance [34]. The protocol defined Evidence Set A for high-dose tigecycline clinical effectiveness, pharmacokinetics or pharmacodynamics, and safety. Evidence Set B covered clinical outcomes, outbreaks, susceptibility, and resistance mechanisms in multidrug-resistant, extensively drug-resistant, or carbapenem-resistant Serratia infection.

Protocol

The protocol, version 1.1 dated July 16, 2026, defined a direct-intersection study as confirmed resistant Serratia infection, a verified intensified tigecycline regimen, and a separately extractable Serratia-specific clinical or microbiological outcome.

Eligibility Criteria

Evidence Set A included adults at least 18 years of age who were critically ill or treated in an ICU-relevant severe-infection setting. Eligible clinical studies used an explicitly reported intensified tigecycline regimen. Single-arm pharmacokinetic and safety cohorts were eligible. Evidence Set B included adult clinical, outbreak, susceptibility, and genomic studies involving resistant Serratia. Reviews, editorials, duplicate reports, animal studies, and laboratory studies unrelated to the defined evidence sets were excluded.

Information Sources and Search Strategy

PubMed/MEDLINE, CENTRAL, and Consensus were searched from inception to July 17, 2026. Searches used controlled vocabulary where available and free-text terms. Evidence Set A combined tigecycline or Tygacil with terms for critical illness, ICU care, sepsis, HAP, VAP, or bloodstream infection and terms for high dose, dose escalation, 200 mg, 100 mg, pharmacokinetics/pharmacodynamics (PK/PD), fibrinogen, or safety. Evidence Set B combined *Serratia marcescens* or Serratia with multidrug-resistant (MDR), extensively drug-resistant (XDR), carbapenem resistance, carbapenem-resistant *Serratia marcescens* (CRSM), carbapenemase, *Klebsiella pneumoniae* carbapenemase (KPC), New Delhi metallo-beta-lactamase (NDM), Verona integron-encoded metallo-β-lactamase (VIM), AmpC, or antimicrobial resistance. Synonyms within each concept were combined with OR, and concept blocks were combined with AND. PubMed used a master search plus a direct-intersection search. CENTRAL used the same concepts through Search Manager. Consensus was a supplementary discovery source. No language restriction was applied. Complete executable strategies and retrieval counts are provided in appendix 1.

Study Selection

Records were deduplicated through automated and manual procedures. Two reviewers and one artificial intelligence system independently screened titles and abstracts. Two reviewers independently assessed full texts and resolved disagreements through discussion. Each excluded report received one principal reason. Studies entered the direct-intersection group only when all organism, resistance, dose, and outcome criteria were met. Linked reports from one study were treated as one study.

Data Extraction and Numerical Verification

Structured forms captured study design, population, infection site, organism, resistance phenotype, tigecycline regimen, companion treatment, raw outcomes, adjusted estimates, PK/PD measures, safety findings, and resistance mechanisms. Missing data were recorded as not reported or not separately extractable. Mixed-organism outcomes were not attributed to Serratia. Published inconsistencies were recorded in appendix 5.

Outcomes

Primary outcomes were author-defined clinical cure or response and microbiological eradication or clearance. Secondary outcomes included mortality, treatment failure, recurrence, and adverse events. PK/PD outcomes included exposure, clearance, distribution, AUC/MIC, and target attainment. Safety outcomes included fibrinogen reduction, coagulation changes, bleeding, and recovery after withdrawal.

Risk of Bias and Methodological Appraisal

The randomized trial was assessed with the revised Cochrane risk-of-bias tool for randomized trials (RoB 2) and non-randomized comparative studies with Risk of Bias in Non-randomized Studies of Interventions (ROBINS-I) [35,36]. Design-appropriate Joanna Briggs Institute (JBI) checklists were used for other clinical and isolate-based designs. JBI results were reported at item level and were not converted into global quality categories.

Synthesis

Studies were synthesized by evidence set, site, organism, dose, outcome, and risk of bias. No meta-analysis was conducted. The direct-intersection group was empty, and the complementary studies were clinically heterogeneous. An exploratory comparison used studies with intravenous tigecycline 200 mg loading, then 100 mg every 12 h versus 100 mg loading, then 50 mg every 12 h. Published n/N values yielded study-level risk ratios with 95% confidence intervals. No pooled effect, study weights, heterogeneity statistic, or overall-effect test were calculated. Mortality was separated by follow-up period.

Results

Study Selection

The searches identified 2,294 records, including 2,165 from PubMed/MEDLINE, 84 from CENTRAL, and 45 from Consensus. Removal of 573 duplicates left 1,721 records for title and abstract screening. Of these, 1,562 were excluded, and 159 reports underwent full-text assessment. A further 123 were excluded because of an ineligible population, outcome, or design (n=68), intervention or exposure (n=26), or comparison group (n=29). A total of 36 primary studies reported in 37 publications were included. The study-selection process is shown in Figure 1 [32].

**Figure 1:**
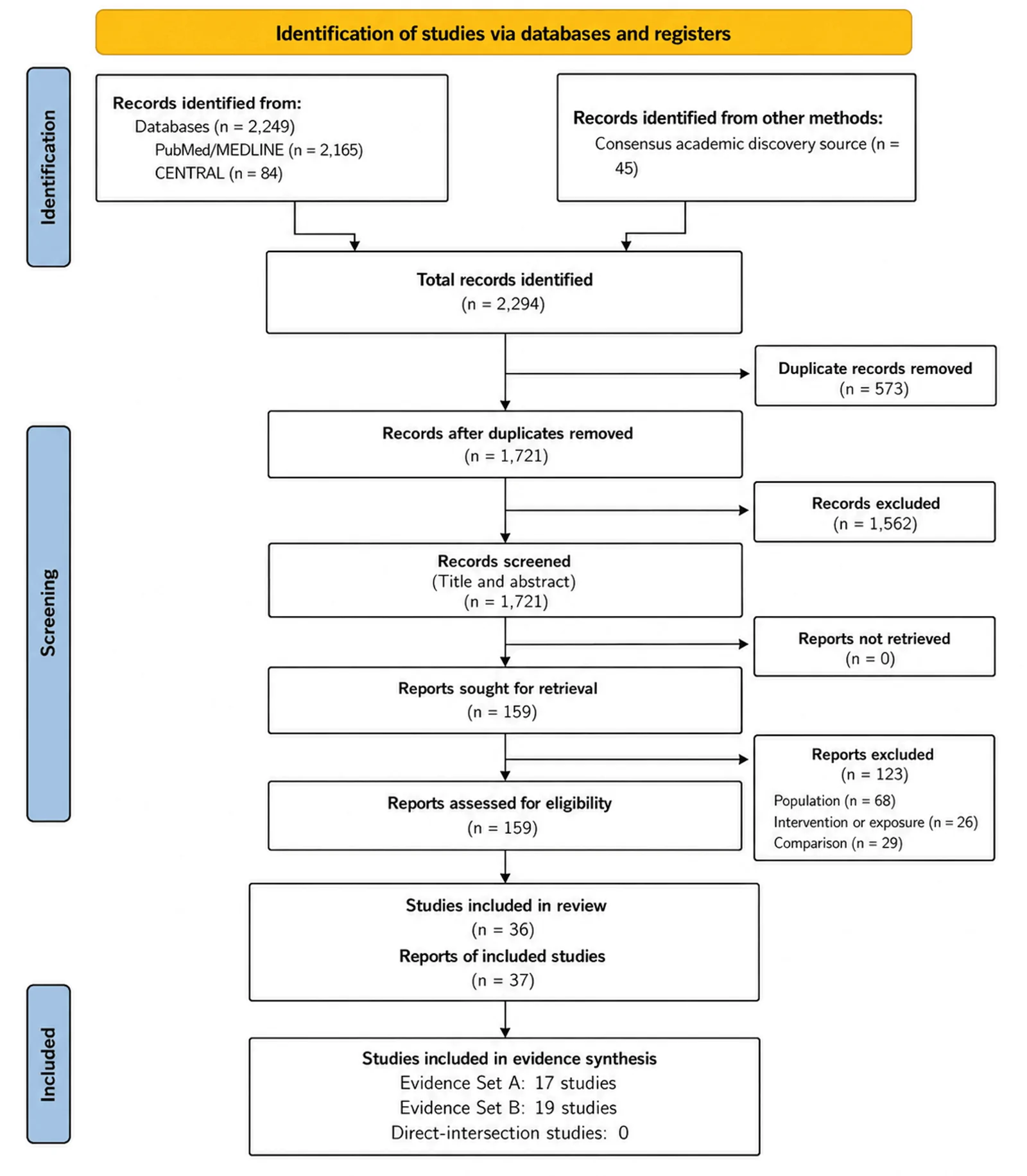
PRISMA 2020 flow diagram of study identification and selection.

Characteristics of the Included Studies

Characteristics of all 36 included studies are summarized in Table 1. Evidence Set A contained 17 studies as follows: nine clinical effectiveness studies [1-7,37,38], four PK/PD studies [8,9,39,40], and four safety studies [10-13]. Evidence Set B contained 19 studies as follows: seven clinical cohorts [14-20], five microbiological or genomic studies [27-29,41,42], and seven outbreak or patient-level studies [21-26,43]. One study had a linked genomic publication [44].

**Table 1:** Characteristics and evidence contribution of all studies included in the review. CRAB: carbapenem-resistant *Acinetobacter baumannii*; CRE: carbapenem-resistant Enterobacterales; HAP: hospital-acquired pneumonia; VAP: ventilator-associated pneumonia; TDM: therapeutic drug monitoring; MDR: multidrug-resistant; XDR: extensively drug-resistant; PK/PD: pharmacokinetics/pharmacodynamics; VIM: Verona integron-encoded metallo-β-lactamase; BSI: bloodstream infection; KPC: *Klebsiella pneumoniae* carbapenemase

Studies	Evidence set	Design	Population/setting	Principal contribution	Relation to target question
Geng et al. (2018) [1]	A1	Retrospective cohort	Critically ill adults; ICU; nosocomial CRKP bloodstream infection	Verified high-dose tigecycline clinical effectiveness	Indirect high-dose evidence
Bai et al. (2020) [2]	A1	Retrospective cohort	Elderly adults; ICU; MDR *Acinetobacter baumannii* pneumonia	Verified high-dose versus standard-dose effectiveness	Indirect high-dose evidence
Han et al. (2021) [3]	A1	Multicenter retrospective cohort	Adults; predominantly ICU; secondary XDR *A. baumannii* bloodstream infection	Verified high-dose versus standard-dose effectiveness	Indirect high-dose evidence
De Pascale et al. (2014) [4]	A1	Retrospective cohort	Critically ill adults; ICU; severe MDR infections	Verified high-dose versus standard-dose effectiveness; includes 4 Serratia isolates without separate outcomes	Indirect high-dose evidence
Ramirez et al. (2013) [5]	A1	Randomized phase 2 trial	Hospitalized adults; HAP/VAP; ICU and non-ICU	High-dose tigecycline efficacy and safety against imipenem-cilastatin	Indirect high-dose evidence
Xia and Jiang (2020) [6]	A1	Retrospective cohort	Elderly adults; ICU and specialist wards; MDR infections	Verified high-dose versus standard-dose effectiveness and safety	Indirect high-dose evidence
Nasomsong et al. (2025) [7]	A1	Propensity score-matched retrospective cohort	Adults; predominantly ICU; CRE bacteremia	High-dose combination-regimen effectiveness	Indirect high-dose evidence
Bai et al. (2023) [37]	A1	Retrospective cohort with TDM	Adults; ICU; carbapenem-resistant Gram-negative pneumonia	Verified high-dose versus standard-dose effectiveness, TDM, and safety	Indirect high-dose evidence
Özgen-Top et al. (2026) [38]	A1	Propensity score-matched retrospective cohort	Adults; HAP/VAP due to CRAB	Verified high-dose versus standard-dose effectiveness and safety	Indirect high-dose evidence
De Pascale et al. (2020) [8]	A2	Prospective PK/PD study	Critically ill adults; ICU; severe infections	High-dose plasma and intrapulmonary pharmacokinetics	Indirect high-dose evidence
Moor et al. (2018) [9]	A2	Prospective population PK study	Adults with sepsis or septic shock	High-dose population pharmacokinetics	Indirect high-dose evidence
Ruiz et al. (2020) [39]	A2	Prospective observational PK/PD study	Critically ill adults; MDR Gram-negative infections	Exposure-target attainment, clinical response, and toxicity	Indirect high-dose evidence
Su et al. (2024) [40]	A2	Prospective population PK study	Critically ill adults; intensive sampling	Population PK and individualized dose simulations	Indirect high-dose evidence
Hu et al. (2020) [10]	A3	Retrospective case-control study	Critically ill adults; ICU	Tigecycline-associated hypofibrinogenemia and bleeding	Indirect high-dose evidence
Routsi et al. (2015) [11]	A3	Observational cohort/research letter	Critically ill adults; ICU	High-dose-associated coagulation changes	Indirect high-dose evidence
Chen and Shi (2018) [12]	A3	Retrospective cohort	Critically ill adults; ICU; VAP due to MDR pathogens	Dose-related adverse events and safety	Indirect high-dose evidence
Treml et al. (2021) [13]	A3	Retrospective cohort	Critically ill adults; ICU	Progression of fibrinogen decrease during high-dose therapy	Indirect high-dose evidence
Zhu et al. (2025) [14]	B1	Retrospective observational cohort	Adults; MDR and carbapenem-resistant Serratia infections	Serratia epidemiology, resistance and clinical outcomes	Serratia-specific context
Cheng et al. (2017) [15]	B1	Propensity score-matched retrospective cohort	Adults; AmpC-producing Enterobacterales bloodstream infection including Serratia	Comparative treatment and mortality outcomes	Serratia-specific context
Lu et al. (2023) [16]	B1	Retrospective comparative cohort	Immunocompromised adults; cefoxitin-non-susceptible Enterobacterales including Serratia	Comparative treatment failure and mortality	Serratia-specific context
Pospišil (2023) [17]	B1	Retrospective cohort	Patients with AmpC-producing Enterobacterales bloodstream infection	Risk factors, treatment, and clinical outcomes	Serratia-specific context
Zilberberg et al. (2014) [18]	B1	Retrospective cohort	Adults with severe sepsis or septic shock due to Gram-negative bacilli	Appropriateness of therapy and hospital outcomes, including Serratia	Serratia-specific context
Leal et al. (2019) [19]	B1	Retrospective observational study	Mixed-age hospital cohort; resistant Gram-negative organisms including Serratia	Resistance epidemiology and outcomes	Serratia-specific context
Slain et al. (2022) [20]	B1	Retrospective single-center cohort	*Serratia marcescens* bacteremia and endocarditis	Antimicrobial treatment patterns and outcomes	Serratia-specific context
Bailey et al. (2026) [21]	B2	Case report with serial genomic analysis	Critically ill adult; KPC-producing Serratia infection	Patient-level treatment course and in vivo resistance evolution	Serratia-specific context
Iovene et al. (2019) [22]	B2	Outbreak report and case series	Three critically ill adults; VIM-producing Serratia	Patient-level treatment and outcomes	Serratia-specific context
Vanni et al. (2023) [23]	B2	ICU outbreak case series	Four critically ill trauma patients; XDR Serratia	Patient-level infection, treatment, and outbreak control	Serratia-specific context
Bes et al. (2021) [24]	B2	Case series	Five carbapenem-resistant Enterobacterales BSIs including one Serratia case	Patient-level treatment and mortality	Serratia-specific context
Tuon et al. (2017) [25]	B2	Hospital outbreak report	Adult ICU and NICU; KPC-producing Serratia	Outbreak epidemiology and adult patient outcomes	Serratia-specific context
da Cruz et al. (2021) [26]/Cruz et al. (2024) [44]	B2	Outbreak report/descriptive case series	Ten patients; KPC-2-producing MDR Serratia; ICU and wards	Clinical outcomes from KPC-2 outbreak with linked whole-genome sequencing report	Serratia-specific context
Wang et al. (2025) [43]	B2	Adult ICU case report with genomic analysis	Adult with carbapenem-resistant Serratia bloodstream infection	Patient-level fatal clinical course and resistance mechanisms	Serratia-specific context
Cosimato et al. (2024) [27]	B3	Retrospective surveillance study	Clinical Serratia isolates	Antimicrobial susceptibility and epidemiological trends	Serratia-specific context
Xu et al. (2024) [28]	B3	Serial-isolate genomic study	Fourteen Serratia isolates from seven patients	Within-host evolution of carbapenem resistance	Serratia-specific context
Jia et al. (2023) [29]	B3	Genomic characterization	Two carbapenem-resistant Serratia bloodstream isolates	Resistance genes, plasmids, and susceptibility	Serratia-specific context
Ataç et al. (2024) [41]	B3	Conference abstract/isolate surveillance	*Serratia marcescens* bloodstream isolates	Susceptibility patterns and carbapenem/aminoglycoside resistance determinants	Serratia-specific context
Ferreira et al. (2020) [42]	B3	Clinical-isolate microbiological and genomic study	ICU/NICU Serratia isolates; adult ICU predominance	KPC, susceptibility, efflux, virulence, and clonality	Serratia-specific context

No study reported confirmed resistant Serratia infection treated with a verified high-dose tigecycline regimen and a separately extractable Serratia-specific outcome. High-dose cohorts contained occasional Serratia isolates without separate results [4,5]. Serratia reports mentioned tigecycline without a verified regimen, and one fatal case involved an isolate with intermediate tigecycline susceptibility but no tigecycline treatment [22-24,26,43].

Evidence Set A1: Clinical Effectiveness of High-Dose Tigecycline

Nine studies assessed clinical effectiveness [1-7,37,38]. High dose usually meant 200 mg loading followed by 100 mg every 12 h. Most participants had HAP, VAP, or bloodstream infection caused by *K. pneumoniae* or *A. baumannii*. No study reported a Serratia-specific effect estimate.

The only randomized trial enrolled 105 treated adults with hospital-acquired pneumonia [5]. Clinically evaluable cure was 17/20 with tigecycline 100 mg every 12 h, 16/23 with 75 mg every 12 h, and 18/24 with imipenem-cilastatin. Death occurred in 3/35, 7/36, and 7/34 patients. A propensity-matched CRE bacteremia study reported better microbiological response with a high-dose-containing regimen, 22/29 versus 12/26, but the adjusted mortality association crossed the null [7].

Observational cohorts showed mixed effects. De Pascale et al. reported higher adjusted clinical cure in VAP (OR: 6.25; 95% CI: 1.59-24.57), but four Serratia isolates had no separate outcomes [4]. Xia and Jiang reported higher clinical response and eradication with similar 28-day mortality [6]. Bai et al. found higher trough exposure without an adjusted dose effect [37]. Özgen-Top et al. found higher microbiological success but no clinical or 30-day survival benefit after matching [38]. Other cohorts showed no consistent mortality advantage [1-3]. Table 1 lists the design, population, and evidence contribution of every included study.

Structured comparison of high-dose versus standard-dose tigecycline studies: Six observational studies used the same verified dose definitions [2-4,6,37,38]. Fixed 28-day or 30-day mortality risk ratios were 0.34 (95% CI: 0.09-1.32), 0.93 (95% CI: 0.66-1.32), 0.91 (95% CI: 0.57-1.44), and 1.30 (95% CI: 0.91-1.85). Every confidence interval crossed 1.0. ICU mortality in De Pascale et al. was RR: 0.73 (95% CI: 0.47-1.12), and mortality before ICU transfer or discharge in Bai et al. was RR: 0.55 (95% CI: 0.14-2.20) [4,37].

Clinical response risk ratios were 1.73 (95% CI: 0.96-3.10), 1.73 (95% CI: 1.22-2.46), 1.48 (95% CI: 1.08-2.02), and 0.84 (95% CI: 0.55-1.28) [4,6,37,38]. Microbiological response risk ratios were 1.08 (95% CI: 0.62-1.90), 1.88 (95% CI: 0.91-3.86), 1.75 (95% CI: 1.09-2.81), 1.64 (95% CI: 0.31-8.71), and 1.92 (95% CI: 1.12-3.28) [2,4,6,37,38]. Outcome definitions and follow-up culture completeness differed.

Because organisms, sites, regimens, mortality periods, and response definitions differed, the estimates were not pooled. Figure 2 displays individual study estimates only and is not a meta-analysis. All six comparisons were observational, and most had serious confounding risk. Table 2 and Figure 2 present the results. No study reported a separately extractable Serratia-specific outcome. The structured study-level comparisons are presented in Table 2 and Figure 2 [2,3,4,6,37,38].

**Figure 2:**
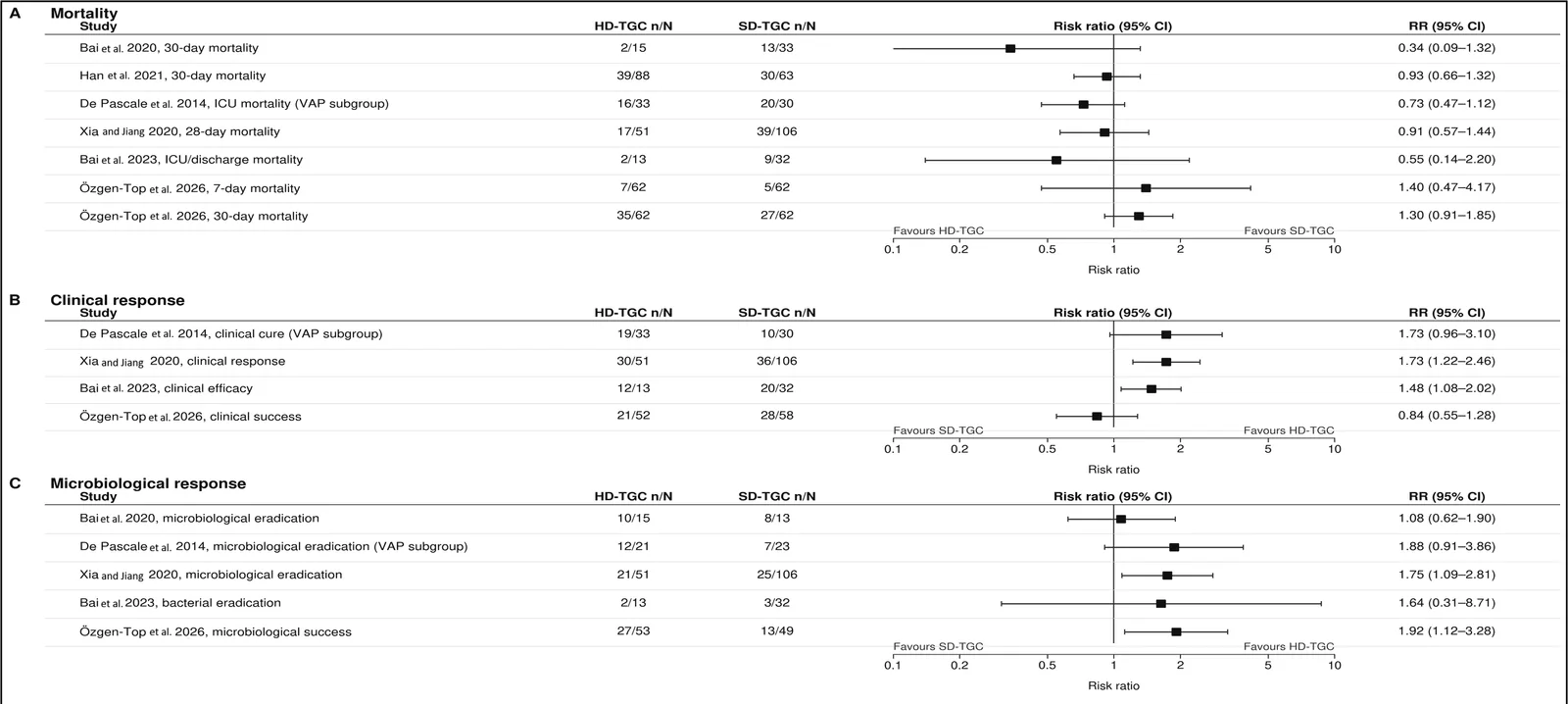
Study-level risk ratios for high-dose versus standard-dose tigecycline without pooling. Squares and horizontal lines show study-level RRs and 95% CIs. Marker size is uniform and does not represent weight. The vertical line marks RR 1.0. No pooled effect, study weights, heterogeneity statistic, or overall-effect test were calculated. HD-TGC: high-dose tigecycline; SD-TGC: standard-dose tigecycline; RR: risk ratio; VAP: ventilator-associated pneumonia

**Table 2:** Structured comparison of high-dose versus standard-dose tigecycline studies. HD-TGC: high-dose tigecycline; SD-TGC: standard-dose tigecycline; RR: risk ratio; MDR: multidrug-resistant; XDR: extensively drug-resistant; HAP: hospital-acquired pneumonia; VAP: ventilator-associated pneumonia; ROBINS-I: Risk of Bias in Non-randomized Studies of Interventions; aOR: adjusted odds ratio; CRAB: carbapenem-resistant *Acinetobacter baumannii*; CR: carbapenem-resistant

Outcome	Study and setting	HD-TGC, n/N	SD-TGC, n/N	Crude RR (95% CI)	Adjusted estimate and appraisal
30-day mortality	Bai et al. (2020) [2]; elderly ICU MDR *A. baumannii *HAP/VAP	2/15	13/33	0.34 (0.09-1.32)	HR: 0.329 (0.074-1.460), p=0.145. ROBINS-I serious
30-day mortality	Han et al. (2021) [3]; XDR *A. baumannii *secondary BSI	39/88	30/63	0.93 (0.66-1.32)	No overall adjusted estimate. ROBINS-I serious
ICU mortality	De Pascale et al. (2014) [4]; mixed MDR VAP	16/33	20/30	0.73 (0.47-1.12)	No adjusted mortality estimate. ROBINS-I serious
28-day mortality	Xia and Jiang (2020) [6]; older adults with mixed MDR infections	17/51	39/106	0.91 (0.57-1.44)	No adjusted mortality estimate. ROBINS-I serious
ICU/discharge mortality	Bai et al. (2023) [37]; CR Gram-negative pneumonia	2/13	9/32	0.55 (0.14-2.20)	No adjusted high-dose estimate. ROBINS-I serious
7-day mortality	Özgen-Top et al. (2026) [38]; CRAB HAP/VAP	7/62	5/62	1.40 (0.47-4.17)	No adjusted 7-day estimate
30-day mortality	Özgen-Top et al. (2026) [38]; CRAB HAP/VAP	35/62	27/62	1.30 (0.91-1.85)	High-dose aOR: 2.83 (0.58-13.7), p=0.196
Clinical cure	De Pascale et al. (2014) [4]; VAP subgroup	19/33	10/30	1.73 (0.96-3.10)	OR: 6.25 (1.59-24.57), p=0.009
Clinical response	Xia and Jiang (2020) [6]; mixed MDR infections	30/51	36/106	1.73 (1.22-2.46)	OR: 5.129 (1.890-13.921), p=0.001
Clinical efficacy	Bai et al. (2023) [37]; CR Gram-negative pneumonia	12/13	20/32	1.48 (1.08-2.02)	No adjusted high-dose estimate; published p=0.070
Clinical success	Özgen-Top et al. (2026) [38]; CRAB HAP/VAP	21/52	28/58	0.84 (0.55-1.28)	Outcome-specific denominators inferred from counts to percentages
Microbiological eradication	Bai et al. (2020) [2]; MDR *A. baumannii *HAP/VAP	10/15	8/13	1.08 (0.62-1.90)	Published direction and p-value were internally inconsistent
Microbiological eradication	De Pascale et al. (2014) [4]; VAP subgroup	12/21	7/23	1.88 (0.91-3.86)	Earlier manuscript RR of 1.75 corrected
Microbiological eradication	Xia and Jiang (2020) [6]; mixed MDR infections	21/51	25/106	1.75 (1.09-2.81)	No adjusted estimate
Bacterial eradication	Bai et al. (2023) [37]; CR Gram-negative pneumonia	2/13	3/32	1.64 (0.31-8.71)	Very small event counts
Microbiological success	Özgen-Top et al. (2026) [38]; CRAB HAP/VAP	27/53	13/49	1.92 (1.12-3.28)	Follow-up cultures were not systematic; serious risk for this outcome

Evidence Set A2: Pharmacokinetic and Pharmacodynamic Findings

Four prospective studies assessed pharmacokinetics or pharmacodynamics [8,9,39,40]. De Pascale et al. reported a median epithelial lining fluid-to-plasma concentration ratio of 152.9% with 200 mg loading, then 100 mg every 12 h [8]. Target attainment declined as MIC or the selected pharmacodynamic target increased. Population models showed wide variability in clearance and distribution [9,40]. Ruiz et al. also found variable exposure, but internal denominator inconsistencies limited its exposure-outcome analysis [39].

These findings support dose-sensitive exposure and show why infection site, MIC, and critical-illness physiology matter. They do not establish clinical effectiveness or define an effective regimen for resistant Serratia. Simulated target attainment cannot be treated as observed treatment success.

Evidence Set A3: Safety and Coagulation

Four dedicated observational studies reported coagulation abnormalities [10-13]. Hu et al. found hypofibrinogenemia in 71/127 patients at a median of six days and normalization in 42/52 evaluable patients after withdrawal [10]. Routsi et al. reported a fall in mean fibrinogen from 563±175 to 289±123 mg/dL by day 14 [11]. Treml et al. found fibrinogen reduction in 59/130 patients and associations with treatment duration or dose per kg [13].

Bai et al. reported a greater mean fibrinogen decline with high dose (-3.05 ± 1.67 versus -1.75 ± 1.90 g/L; p=0.038) [37]. Özgen-Top et al. reported more hepatic and platelet abnormalities with high dose but no difference in treatment withdrawal [38]. Sepsis, hepatic dysfunction, and concurrent drugs were competing causes. Baseline and serial coagulation assessment is reasonable during high-dose or prolonged treatment, but no universal testing interval can be defined. The pharmacokinetic, pharmacodynamic, and safety findings are summarized in Table 3.

**Table 3:** Pharmacokinetic, pharmacodynamic, and safety findings in Evidence Sets A2 and A3. ELF: epithelial lining fluid; MIC: minimum inhibitory concentration; HD: high-dose; SD: standard-dose; PK/PD: pharmacokinetics/pharmacodynamics; CL: clearance; V1: volume of distribution in the central compartment; V2: volume of distribution in the peripheral compartment; VAP: ventilator-associated pneumonia

Domains/studies	Design and sample	Exposure	Principal findings	Limitation and interpretation
A2: De Pascale et al. (2020) [8]	Prospective ICU PK/PD; n=32	200 mg loading, then 100 mg q12h	Median ELF/plasma ratio 152.9%. Target attainment fell as MIC and selected target increased	Non-Serratia cohort. Exposure evidence only
A2: Moor et al. (2018) [9]	Prospective population PK; n=37 with sepsis or septic shock	200 mg loading then 100 mg q12h	Two-compartment model with interindividual variability in clearance and distribution	Short sampling period and no clinical comparator
A2: Ruiz et al. (2020) [39]	Prospective ICU PK/PD; n=25	HD n=15 versus SD n=10	Higher exposure with HD. Thirty-day mortality 9/25; hepatobiliary events 8/25. Published target-attainment totals were inconsistent	Small, heterogeneous cohort and overfitted analyses. PK/PD evidence only
A2: Su et al. (2024) [40]	Prospective population PK; n=98; 751 concentrations	91 received 100 mg/day; 7 received 200 mg/day	CL: 3.09 L/h; V1: 32.1 L; V2: 113 L. Simulations linked target attainment to MIC, target, and covariates	Predominantly standard dose and model-based recommendations without comparative clinical outcomes
A3: Hu et al. (2020) [10]	Retrospective cohort; n=127 critically ill patients	Dose and duration assessed	Hypofibrinogenemia 71/127; median onset 6 days. Fibrinogen normalized in 42/52 evaluable patients after withdrawal	Observational safety signal with competing causes
A3: Routsi et al. (2015) [11]	Observational ICU cohort; n=45	High-dose tigecycline	Mean fibrinogen fell from 563±175 mg/dL to 289±123 mg/dL by day 14; p<0.001	Small descriptive cohort
A3: Chen and Shi (2018) [12]	Retrospective VAP cohort; n=92	HD, SD, and low-dose groups	Hepatic and coagulation laboratory changes were reported across dose groups	Severe bleeding, transfusion, and discontinuation were incompletely reported
A3: Treml et al. (2021) [13]	Retrospective ICU cohort; n=130	Dose, duration, and cumulative exposure assessed	Fibrinogen reduction in 59/130. Lower weight, duration, and dose per kg were associated with reduction	Retrospective exposure-outcome analysis

Evidence Set B1: Serratia Clinical Cohorts

Seven clinical cohorts described resistant Serratia treatment or outcomes [14-20]. Tigecycline was not evaluated as a defined active regimen. Zhu et al. reported 28-day mortality of 4/14 in carbapenem-resistant infection [14]. Mixed AmpC bloodstream-infection cohorts included 45, 22, and 47 Serratia cases but did not provide organism-specific comparative effects [15-17].

A broader severe sepsis cohort initially linked active treatment to better outcomes [18]. Leal et al. reported 15 Serratia isolates without organism-specific mortality [19]. Slain et al. described 43 adults with bacteremia or endocarditis in small non-randomized treatment groups [20]. These studies describe resistance burden and treatment context but do not rank regimens for Serratia. The clinical cohort findings, clinical treatment, and outcome evidence for resistant Serratia infections are summarized in Table 4.

**Table 4:** Clinical treatment and outcome evidence for MDR/XDR/CR Serratia infections in Evidence Set B1. MDR: multidrug-resistant; XDR: extensively drug-resistant; CR: carbapenem-resistant; CRSM: carbapenem-resistant *Serratia marcescens*; BSI: bloodstream infection; ROBINS-I: Risk of Bias in Non-randomized Studies of Interventions

Studies	Population and Serratia contribution	Treatment comparison/exposure	Key outcomes	Use in synthesis
Zhu et al. (2025) [14]	n=242 *S. marcescens*; 31 MDR and 14 CRSM	Clinically selected regimens	CRSM 28-day mortality 4/14. Prior hospitalization associated with BSI	Direct Serratia epidemiology and resistance burden
Cheng et al. (2017) [15]	n=165 AmpC BSI; 45 Serratia; 41 matched pairs	Piperacillin-tazobactam versus cefepime/meropenem	Mortality 6/41 versus 3/41; persistent bacteremia 8/41 versus 4/41	Moderate ROBINS-I risk; pooled organisms
Lu et al. (2023) [16]	n=81 immunocompromised adults; 22 Serratia	Piperacillin-tazobactam versus cefepime/carbapenem	Failure 17/35 versus 17/46. Early microbiological failure 4/35 versus 0/46; p=0.019.	Serious ROBINS-I risk; no Serratia-specific effect
Pospišil et al. (2023) [17]	n=172 AmpC BSI; 47 Serratia	Mixed beta-lactam regimens	Clinical cure 78.52% in mixed cohort. Resistant versus susceptible mortality: 13/54 versus 24/118	Descriptive mixed-organism evidence
Zilberberg et al. (2014) [18]	n=1,076 Gram-negative severe sepsis; 46 Serratia	Appropriate versus inappropriate initial therapy	Appropriate initial therapy in 39/46 Serratia cases	Early active-treatment context only
Leal et al. (2019) [19]	n=143 patients; 15 Serratia isolates	Empirical and culture-guided regimens	One Serratia isolate was MDR, and 14 were non-MDR. Serratia mortality not reported	Epidemiological context only
Slain et al. (2022) [20]	n=43 Serratia bacteremia/endocarditis	Cefepime, meropenem, piperacillin-tazobactam and combinations	Cefepime 13/19 cures; meropenem 3/6; piperacillin-tazobactam 3/5	Small, non-randomized groups

Evidence Set B2: Outbreak and Patient-Level Reports

Seven studies described outbreaks or individual patients [21-26,43]. Reports included KPC, VIM, NDM, and mixed carbapenemase phenotypes. Tigecycline was mentioned in several fatal cases, but dose, duration, or companion-drug activity was not adequately reported [22-24,26]. Adult ICU mortality was 2/7 in one KPC-2 outbreak [25]. A Brazilian outbreak reported four deaths among 10 patients and had a linked genomic report [26,44].

Wang et al. described a fatal adult bloodstream infection caused by a strain carrying blaNDM-1, blaKPC-2, and blaSRT-2 [43]. The tigecycline minimum inhibitory concentration (MIC) was intermediate, and the patient did not receive tigecycline. Patient-level reports therefore provide clinical and resistance context but cannot estimate the effectiveness or harm of high-dose tigecycline. Patient-level and outbreak evidence is summarized in appendix 2.

Evidence Set B3: Susceptibility and Genomic Findings

Surveillance and genomic studies showed marked variation in susceptibility and resistance mechanisms [27-29,41,42]. Cosimato et al. reported variable carbapenem and cephalosporin resistance across 396 isolates [27]. Ataç et al. found three carbapenem-resistant isolates among 98 bloodstream isolates and one blaOXA-48-positive strain [41]. Ferreira et al. reported tigecycline non-susceptibility in 50/54 KPC-producing isolates [42].

Serial-isolate and genomic studies documented acquisition or increased expression of blaKPC, KPC-2 IncR plasmids, and transferable NDM-1 or KPC-2 plasmids [28,29,43,44]. These findings support repeat susceptibility testing when response is poor. They do not provide a Serratia-specific tigecycline breakpoint or show that in vitro activity produces clinical success. Microbiological and genomic evidence is summarized in appendix 3.

Cross-Evidence Interpretation

Evidence Set A described high-dose tigecycline mainly in *K. pneumoniae* or *A. baumannii*, whereas Evidence Set B described Serratia outcomes and resistance biology. Differences in organism, site, and design prevented transferability. PK/PD evidence provides limited dosing rationale at favorable MICs [8,9,39,40], and safety studies identify a repeated coagulation signal [10-13,37,38].

Risk of Bias and Reporting Inconsistencies

The randomized trial had some concerns because it stopped early [5]. Propensity-matched studies had moderate risk for selected outcomes [7,15,38], and most unmatched comparative cohorts had serious confounding risk [1-4,6,16,37]. The numerical audit identified inconsistencies in eight studies [2,6,7,37-40,43]. Appendix 4 provides the methodological appraisal, and appendix 5 lists numerical inconsistencies.

Discussion

Principal Findings

No verified study directly evaluated high-dose tigecycline in resistant Serratia infection with a separately extractable organism-specific outcome. Indirect evidence showed possible improvement in clinical response or culture clearance in selected severe multidrug-resistant Gram-negative infections but no consistent survival benefit. Resistant Serratia studies assessed other treatments or resistance mechanisms rather than a defined high-dose tigecycline regimen.

Separating the evidence sets prevented results from *K. pneumoniae* or *A. baumannii* being assigned to Serratia. Unverified tigecycline exposure in fatal cases cannot be labeled treatment failure because severity, coinfection, source control, and companion-drug activity were not controlled. The review therefore defines an evidence boundary rather than an organism-specific treatment estimate.

Clinical Effectiveness and Pharmacokinetic Interpretation

Clinical and microbiological response signals were stronger than mortality signals. The randomized trial was small and stopped early [5]. A propensity-matched carbapenem-resistant Enterobacterales (CRE) bacteremia study found better microbiological response but no precise adjusted mortality effect [7]. The six-study comparison found no precise fixed-time mortality benefit [2-4,6,37,38]. Heterogeneous definitions and serious confounding prevented a common effect estimate.

PK/PD studies showed dose-sensitive exposure and declining target attainment as MIC increased [8,9,39,40]. High epithelial lining fluid exposure supports biological plausibility for pneumonia but cannot be transferred to bloodstream infection. Model-based target attainment did not prove clinical success or define an effective Serratia regimen.

Safety and Coagulation Effects

Fibrinogen reduction was reported in dedicated safety cohorts and comparative studies [10-13,37,38]. Associations with dose, duration, or cumulative exposure and recovery after withdrawal support a treatment-related component. Baseline and serial coagulation assessment is reasonable during high-dose or prolonged therapy, interpreted alongside sepsis, hepatic function, and concurrent drugs.

Serratia Evidence and Clinical Implications

Serratia cohorts and mixed AmpC studies provide treatment context but no organism-specific ranking of cefepime, carbapenems, piperacillin-tazobactam, or tigecycline [14-20]. Outbreak and genomic reports documented carbapenemases, plasmid spread, and within-host resistance evolution [21-29,41-44]. These findings support repeat susceptibility assessment when response is poor. A recent resistance review supplied no direct high-dose clinical outcome data [45].

Current evidence does not support a Serratia-specific high-dose tigecycline recommendation. Decisions should consider infection site, susceptibility, source control, and active alternatives. Bloodstream infection needs caution because plasma exposure is limited and target attainment declines as MIC increases [8,9,39,40]. Coagulation monitoring should be considered for high doses or longer courses [10-13,37,38]. Lack of response should prompt reassessment rather than automatic dose escalation [21,28].

Strengths, Limitations, and Research Priorities

This review preserved the organism-treatment question, separated indirect evidence, and verified study-level data. Design-specific appraisal scales were retained, and published inconsistencies were disclosed. Limitations include the absence of direct studies, retrospective designs, and missing dose- or organism-specific outcomes. Searches covered PubMed/MEDLINE, CENTRAL, and Consensus but not Embase, Scopus, Web of Science, trial registries, or conference databases. One study was abstract-only, and publication bias could not be assessed.

Future studies should report Serratia outcomes separately with exact dose, site, source control, MIC method, and active companion drugs. PK/PD studies should link unbound exposure to Serratia-specific MICs and clinical outcomes.

## Conclusions

High-dose tigecycline efficacy for multidrug-resistant or carbapenem-resistant Serratia infection is not established. Indirect evidence shows possible clinical or microbiological response benefits in selected severe Gram-negative infections but no consistent survival advantage. PK/PD findings provide biological plausibility rather than clinical proof. Treatment should be individualized by infection site, susceptibility, source control, and active alternatives, with coagulation monitoring during high-dose or prolonged therapy.

## Appendices

Appendix 1

**Table 5:** Search strategies and retrieval log.

Source and platform	Coverage	Date searched	Search purpose	Strategy location	Limits	Records retrieved
PubMed/MEDLINE	Database inception to search date	July 17, 2026	Combined Evidence Sets A and B master search	Appendix 1	No language limit	2,078
PubMed/MEDLINE	Database inception to search date	July 17, 2026	Direct-intersection validation search	PubMed/MEDLINE Master Search	No language limit	87
CENTRAL Search Manager	Database inception to search date	July 17, 2026	Combined evidence sets A and B, line #23	CENTRAL Search Manager strategy	Trials records	84
CENTRAL Search Manager	Database inception to search date	July 17, 2026	Tigecycline AND Serratia validation line	CENTRAL Search Manager strategy	Trials records	0; validation only
Consensus	Indexed peer-reviewed literature	July 17, 2026	Evidence set A query	Consensus Supplementary Searches	Displayed report references	16
Consensus	Indexed peer-reviewed literature	July 17, 2026	Evidence set B query	Consensus Supplementary Searches	Displayed report references	16
Consensus	Indexed peer-reviewed literature	July 17, 2026	Direct-intersection query	Consensus Supplementary Searches	Displayed report references	13

PubMed/MEDLINE Master Search

((tigecycline[Title/Abstract] OR Tygacil[Title/Abstract]) AND ((critically ill[Title/Abstract] OR critical illness[Title/Abstract] OR intensive care[Title/Abstract] OR ICU[Title/Abstract] OR sepsis[Title/Abstract] OR septic shock[Title/Abstract] OR severe infection*[Title/Abstract] OR ventilator-associated pneumonia[Title/Abstract] OR VAP[Title/Abstract] OR hospital-acquired pneumonia[Title/Abstract] OR HAP[Title/Abstract] OR bloodstream infection*[Title/Abstract] OR bacteremia[Title/Abstract] OR bacteraemia[Title/Abstract]) AND (high dose[Title/Abstract] OR high-dose[Title/Abstract] OR higher dose[Title/Abstract] OR higher-dose[Title/Abstract] OR high dosage[Title/Abstract] OR dose escalation[Title/Abstract] OR 200 mg[Title/Abstract] OR 100 mg[Title/Abstract] OR pharmacokinetic*[Title/Abstract] OR pharmacodynamic*[Title/Abstract] OR PKPD[Title/Abstract] OR fibrinogen*[Title/Abstract] OR hypofibrinogen*[Title/Abstract] OR coagul*[Title/Abstract] OR adverse event*[Title/Abstract] OR safety[Title/Abstract]))) OR (((*Serratia marcescens*[Title/Abstract]) OR Serratia[Title/Abstract] OR Serratia[MeSH Terms]) AND (multidrug resistant[Title/Abstract] OR multidrug-resistant[Title/Abstract] OR MDR[Title/Abstract] OR extensively drug resistant[Title/Abstract] OR extensively-drug-resistant[Title/Abstract] OR XDR[Title/Abstract] OR carbapenem resistant[Title/Abstract] OR carbapenem-resistant[Title/Abstract] OR CRSM[Title/Abstract] OR carbapenemase*[Title/Abstract] OR KPC[Title/Abstract] OR NDM[Title/Abstract] OR VIM[Title/Abstract] OR AmpC[Title/Abstract] OR antimicrobial resistance[Title/Abstract] OR antibiotic resistance[Title/Abstract])))

A total of 2,078 records were retrieved, with no language restrictions applied.

PubMed/MEDLINE Direct-Intersection Validation Search

((tigecycline[Title/Abstract] OR Tygacil[Title/Abstract]) AND ((*Serratia marcescens*[Title/Abstract]) OR Serratia[Title/Abstract] OR Serratia[MeSH Terms]))

A total of 87 records were retrieved as a validation set and cross-checked against the master search records before deduplication.

CENTRAL Search Manager Strategy

#1 MESH DESCRIPTOR Tigecycline EXPLODE ALL TREES, #2 (tigecycline OR Tygacil):ti,ab,kw, #3 #1 OR #2, #4 MeSH descriptor Critical Illness explode all trees, #5 MeSH descriptor Intensive Care Units explode all trees, #6 MeSH descriptor Sepsis explode all trees, #7 MeSH descriptor Shock, Septic explode all trees, #8 MeSH descriptor Pneumonia, Ventilator-Associated explode all trees, #9 MeSH descriptor Bacteremia explode all trees, #10 ("critically ill" OR "critical illness" OR "intensive care" OR ICU OR sepsis OR "septic shock" OR "severe infection" OR "severe infections" OR "ventilator-associated pneumonia" OR VAP OR "hospital-acquired pneumonia" OR HAP OR "bloodstream infection" OR "bloodstream infections" OR bacteremia OR bacteraemia):ti,ab,kw, #11 #4 OR #5 OR #6 OR #7 OR #8 OR #9 OR #10, #12 ("high dose" OR "high-dose" OR "higher dose" OR "higher-dose" OR "high dosage" OR "high-dosage" OR "dose escalation" OR "200 mg" OR "100 mg" OR pharmacokinetic* OR pharmacodynamic* OR PKPD OR fibrinogen* OR hypofibrinogen* OR coagul* OR "adverse event" OR "adverse events" OR safety):ti,ab,kw, #13 #3 AND #11 AND #12, #14 MeSH descriptor *Serratia marcescens* explode all trees, #15 ("*Serratia marcescens*" OR Serratia):ti,ab,kw, #16 #14 OR #15, #17 MeSH descriptor Drug Resistance, Multiple, Bacterial explode all trees, #18 MeSH descriptor Drug Resistance, Bacterial explode all trees, #19 MeSH descriptor beta-Lactamases explode all trees, #20 ("multidrug resistant" OR "multidrug-resistant" OR MDR OR "extensively drug resistant" OR "extensively-drug-resistant" OR XDR OR "carbapenem resistant" OR "carbapenem-resistant" OR CRSM OR carbapenemase* OR KPC OR NDM OR VIM OR AmpC OR "antimicrobial resistance" OR "antibiotic resistance"):ti,ab,kw, #21 #17 OR #18 OR #19 OR #20, #22 #16 AND #21, and #23 #13 OR #22

Line #23 retrieved 84 records. The validation line #3 AND #16 retrieved 0 records and was not added to the identification total.

Consensus Supplementary Searches

The Evidence Set A query focused on high-dose tigecycline in critically ill or intensive care patients with multidrug-resistant bacterial infections, including clinical outcomes, mortality, microbiological response, pharmacokinetics, and safety. The exported report contained 16 references.

The Evidence Set B query focused on multidrug-resistant, extensively drug-resistant, or carbapenem-resistant *Serratia marcescens *infections, including clinical treatment, bloodstream infections, intensive care, outbreaks, and antimicrobial resistance. The exported report contained 16 references.

The direct-intersection query focused on high-dose tigecycline treatment for *Serratia marcescens* infections in critically ill or intensive care patients. The exported report contained 13 references.

Consensus was treated as a supplementary academic discovery source, with diligent validation of DOIs against the actual sources of publications. The three reports contributed 45 listed references before deduplication.

Appendix 2

**Table 6:** Serratia outbreak and patient-level evidence in Evidence Set B2. Tigecycline exposure without a reported loading and maintenance dose does not qualify as verified high-dose treatment. The Cruz et al. (2024) genomic report is linked to the 2021 outbreak study and is not counted as a separate study. MDR: multidrug-resistant; XDR: extensively drug-resistant; VIM: Verona integron-encoded metallo-β-lactamase; KPC: *Klebsiella pneumoniae* carbapenemase; NDM: New Delhi metallo-beta-lactamase; CRE: carbapenem-resistant Enterobacterales; BSI: bloodstream infection; MIC: minimum inhibitory concentration; CRSM: carbapenem-resistant *Serratia marcescens*

Study	Patients/setting	Resistance or organism	Tigecycline exposure	Outcome	Interpretive status
Bailey et al. (2026) [21]	One adult liver-transplant recipient	KPC-producing *S. marcescens* with KPC-44 evolution	None reported	Fatal course	Resistance-evolution context only
Iovene et al. (2019) [22]	Three adult ICU patients	VIM-producing *S. marcescens*	One patient received tigecycline; dose and duration unreported	One survived and two died	No verified high-dose regimen
Vanni et al. (2023) [23]	Four adult trauma ICU patients	XDR NDM-producing *S. marcescens*	One patient received meropenem plus tigecycline; regimen unreported	Three survived and one died	Outbreak evidence; colonization separated
Bes et al. (2021) [24]	Five CRE BSI cases; one Serratia	NDM-1 and KPC-2 co-producing *S. marcescens*	Tigecycline plus fosfomycin; regimen unreported	Serratia patient died after 11 days	Single case and no causal inference
Tuon et al. (2017) [25]	Seven adult ICU and six NICU patients	KPC-2-producing *S. marcescens*	No clinical tigecycline treatment established	Adult cohort: two deaths and five survivors	Adult and neonatal data separated
da Cruz et al. (2021) [26] with Cruz et al. (2024) [44]	Ten patients in ICU and wards	KPC-2-producing MDR *S. marcescens*	Gentamicin plus tigecycline in three patients; doses unreported	Four deaths and six cures/resolutions	One study with linked clinical and genomic publications
Wang et al. (2025) [43]	One 96-year-old ICU patient	CRSM with blaNDM-1, blaKPC-2 and blaSRT-2	Tigecycline was not administered; MIC 4 mg/L was classified as intermediate	Recurrent BSI, multiple-organ failure, and death	Patient-level and plasmid context only

Appendix 3

Microbiological, Susceptibility, and Genomic Evidence in Evidence Set B3

**Table 7:** Microbiological, susceptibility, and genomic evidence in Evidence Set B3. MIC: minimum inhibitory concentration

Studies	Material and sample	Principal susceptibility/genomic findings	Clinical inference permitted
Cosimato et al. 2024 [27]	396 clinical isolates, 2018-2022	Meropenem and ceftazidime-avibactam resistance was uncommon in the surveillance series; resistance varied by year	Local epidemiology only
Xu et al. (2024) [28]	14 serial isolates from 7 patients	Meropenem MIC increased eight- to 1,024-fold through blaKPC acquisition or expression and porin-related changes	Within-host resistance evolution only
Jia et al. (2023) [29]	Two carbapenem-resistant bloodstream isolates	KPC-2-encoding IncR plasmids and other resistance determinants	Genomic context only
Ataç et al. (2024) [41]	98 bloodstream isolates; abstract only	Three carbapenem-resistant isolates; one blaOXA-48 positive. Broad susceptibility remained high for several agents	Susceptibility context only
Ferreira et al. (2020) [42]	54 isolates from 45 ICU or NICU patients	Tigecycline non-susceptibility: 50/54; colistin non-susceptibility: 52/54; blaKPC and blaTEM in all isolates; major clonal cluster: 52/54	Resistance and clonality context only

Appendix 4

Study-Level Methodological Appraisal and Role in Synthesis

**Table 8:** Comparative intervention studies assessed with RoB 2 or ROBINS-I. RoB 2 and ROBINS-I results are reported using the overall judgment categories defined by each tool. ROBINS-I judgments are outcome-specific where risk differed across outcomes. JBI results were reported at item level and were not converted into scores, percentages, or low, moderate, or high categories. Adapted appraisal means that the closest JBI checklist was used to assess reporting and methodological features of isolate-based surveillance or genomic studies. It was not used as a clinical treatment-effect risk-of-bias tool. Results from different tools were not combined into one quality scale. JBI: Joanna Briggs Institute; BSI: bloodstream infection; ROBINS-I: Risk of Bias in Non-randomized Studies of Interventions; RoB 2: revised Cochrane risk-of-bias tool for randomized trials

Studies	Evidence set	Appraisal tool	Overall judgment	Principal limitation	Role in synthesis
Geng et al. (2018) [1]	A1	ROBINS-I	Serious	Confounding by indication and small cohort	Comparative effect interpreted cautiously
Bai et al. (2020) [2]	A1	ROBINS-I	Serious	Confounding, small groups, and reporting inconsistencies	Comparative effect interpreted cautiously
Han et al. (2021) [3]	A1	ROBINS-I	Serious	Residual confounding and subgroup dependence	Subgroup findings interpreted cautiously
De Pascale et al. (2014) [4]	A1	ROBINS-I	Serious	Clinician-selected dose and retrospective design	Supportive non-randomized evidence
Ramirez et al. (2013) [5]	A1	RoB 2	Some concerns	Early termination and missing clinically evaluable outcomes	Randomized evidence with limited precision
Xia and Jiang (2020) [6]	A1	ROBINS-I	Serious	Baseline imbalance and inconsistent published estimate	Comparative effect interpreted cautiously
Nasomsong et al. (2025) [7]	A1	ROBINS-I	Moderate	Small matched sample and incomplete cultures	Adjusted comparison; no Serratia cases
Bai et al. (2023) [37]	A1	ROBINS-I	Serious	Physician-selected dose, small groups, and reporting inconsistencies	Supportive comparative evidence
Özgen-Top et al. (2026) [38]	A1	ROBINS-I	Moderate for mortality; serious for microbiology	Five-day eligibility and non-systematic follow-up cultures	Mortality comparison and cautious microbiology interpretation
Cheng et al. (2017) [15]	B1	ROBINS-I	Moderate	Small propensity-matched sample	Mixed-organism AmpC BSI comparison
Lu et al. (2023) [16]	B1	ROBINS-I	Serious	Baseline imbalance and residual confounding	Comparative effect interpreted cautiously

**Table 9:** Descriptive, non-comparative, and isolate-based studies assessed with JBI checklists. JBI results were reported at item level and were not converted into scores, percentages, or low, moderate, or high categories. Adapted appraisal means that the closest JBI checklist was used to assess reporting and methodological features of isolate-based surveillance or genomic studies. It was not used as a clinical treatment-effect risk-of-bias tool. Results from different tools were not combined into one quality scale. JBI: Joanna Briggs Institute; N/A: not applicable; PK/PD: pharmacokinetics/pharmacodynamics; PTA: probability of target attainment

Studies	Evidence set	Appraisal tool	Item-level result	Principal limitation	Role in synthesis
De Pascale et al. (2020) [8]	A2	JBI cohort	Q1 N/A; Q4 and Q5 not met; other items met	Small single-arm PK/PD cohort	PK/PD evidence only
Moor et al. (2018) [9]	A2	JBI cohort	Q1 N/A; Q4 and Q5 not met; other items met	Short sampling period and no comparator	Population PK only
Ruiz et al. (2020) [39]	A2	JBI cohort	Q1-Q8 met; Q9 unclear; Q10 and Q11 not met	Small cohort, overfitted models, and inconsistent denominators	PK/PD and toxicity evidence
Su et al. (2024) [40]	A2	JBI cohort	Q1, Q6, and Q10 N/A; all applicable items met	Mostly standard dose and simulation-based recommendations	Population PK and PTA evidence
Hu et al. (2020) [10]	A3	JBI cohort	All 11 items met	Retrospective safety analysis	Safety signal
Routsi et al. (2015) [11]	A3	JBI cohort	Q5 not met; other items met	Small descriptive cohort	Safety signal
Chen and Shi (2018) [12]	A3	JBI cohort	All 11 items met	Incomplete treatment-modification reporting	Safety context
Treml et al. (2021) [13]	A3	JBI cohort	All 11 items met	Retrospective exposure-outcome analysis	Dose and duration safety context
Zhu et al. (2025) [14]	B1	JBI cohort	All 11 items met	Single-center retrospective study	Serratia epidemiology
Pospišil et al. (2023) [17]	B1	JBI cohort	Q5 not met; other items met	No adjusted treatment-effect analysis	Descriptive outcome context
Zilberberg et al. (2014) [18]	B1	JBI cohort	All 11 items met	Mixed-organism outcomes	Appropriate-treatment context
Leal et al. (2019) [19]	B1	JBI cohort	All 11 items met	Serratia-specific outcomes not extractable	Epidemiological context
Slain et al. (2022) [20]	B1	JBI cohort	Q5 not met; other items met	Small unadjusted treatment groups	Serratia treatment context
Bailey et al. (2026) [21]	B2	JBI case report	All 8 items met	Single fatal case	Resistance-evolution context
Iovene et al. (2019) [22]	B2	JBI case series	Q1-Q9 met; Q10 N/A	Three-patient outbreak	Outbreak context
Vanni et al. (2023) [23]	B2	JBI case series	Q1-Q9 met; Q10 N/A	Four-patient outbreak	Outbreak context
Bes et al. (2021) [24]	B2	JBI case series	Q1-Q9 met; Q10 N/A	One Serratia patient in five cases	Patient-level context
Tuon et al. (2017) [25]	B2	JBI case series	Q1-Q9 met; Q10 N/A	Adult and neonatal data	Outbreak context
da Cruz et al. (2021) [26]	B2	JBI case series	Q1-Q9 met; Q10 N/A	Unreported tigecycline doses and linked genomic report	Outbreak and genomic context
Wang et al. (2025) [43]	B2	JBI case report	All 8 items met	Single fatal case; no tigecycline treatment	Patient-level and genomic context
Cosimato et al. (2024) [27]	B3	Adapted JBI cross-sectional	Q1-Q4, Q7 and Q8 met; Q5 and Q6 not met	Local isolate surveillance	Microbiological context
Xu et al. (2024) [28]	B3	Adapted JBI case series	Q1-Q9 met; Q10 N/A	Serial isolates from seven patients	Genomic evolution context
Jia et al. (2023) [29]	B3	Adapted JBI cross-sectional	Q1-Q4 and Q7 met; Q5-Q6 not met; Q8 N/A	Two-isolate genomic description	Genomic context
Ataç et al. (2024) [41]	B3	Adapted JBI cross-sectional	Q1, Q3, Q4, Q7 and Q8 met; Q2 unclear; Q5-Q6 not met	Abstract-only isolate surveillance	Susceptibility context
Ferreira et al. (2020) [42]	B3	Adapted JBI cross-sectional	Q1-Q4, Q7 and Q8 met; Q5-Q6 not met	Mixed ICU/NICU and surveillance isolates	Resistance and clonality context

Appendix 5

**Table 10:** Author-reported inconsistencies and extraction corrections requiring annotation. APACHE II: Acute Physiology and Chronic Health Evaluation II; MDR: multidrug-resistant; RR: risk ratio; PK: pharmacokinetics; MIC: minimum inhibitory concentration

Studies/item	Published or extracted inconsistency	Final handling
Nasomsong et al. (2025) [7]	Comparator mortality reported as 21/37 (71.4%); the mathematical value is 56.8%	Retain n/N, state both percentages, and preserve HR
Xia and Jiang (2020) [6]	APACHE II OR (0.048) lies outside 95% CI (0.179-0.926)	Identify as author reporting error and do not interpret
Bai et al. (2020) [2]	Abstract reverses eradication direction; p=0.02 is not reproducible; 2/15 was printed as 14.0%	Use n/N and annotate author-reported values
Leal et al. (2019) [19]	Values 1 and 14 were MDR and non-MDR isolates, not deaths and survivors	Report Serratia-specific mortality as not reported
Chen and Shi (2018) [12]	A treatment-discontinuation statement was copied from another study during earlier extraction	Remove statement and record not reported
De Pascale et al. (2014) [4]	Earlier manuscript microbiological RR was 1.75, but 12/21 versus 7/23 gives 1.88	Use RR 1.88 (95% CI: 0.91-3.86)
Bai et al. (2023) [37]	Study period conflicts between Abstract and Methods; one univariate OR lies outside its CI	Use the Methods period and adjusted estimate only
Özgen-Top et al. (2026) [38]	Bilirubin counts and p-values conflict between the text and Table 5; microbiological p differs in the Discussion	Use Table 5 and Table 2 values with annotation
Özgen-Top et al. (2026) [38]	Outcome-specific clinical and microbiological denominators were not printed	Infer denominators exactly from counts and percentages and footnote
Ruiz et al. (2020) [39]	PK total n, target-attainment total, and treatment-failure denominator conflict	Do not calculate a dose effect from inconsistent totals
Su et al. (2024) [40]	One bootstrap CI does not contain its parameter estimate	Do not quote the inconsistent CI
Wang et al. (2025) [43]	Abstract wording implies tigecycline exception, but Table 1 classifies MIC 4 mg/L as intermediate	Use intermediate and state no tigecycline treatment
